# A Dramatic Change in Rheological Behavior of a Clay Material Caused by a Minor Addition of Hydrophilic and Amphiphilic Polyelectrolytes

**DOI:** 10.3390/polym13213662

**Published:** 2021-10-24

**Authors:** Irina G. Panova, Alexander A. Kiushov, Dolgor D. Khaydapova, Sergey B. Zezin, Maxim S. Arzhakov, Alexander A. Yaroslavov

**Affiliations:** 1 Department of Chemistry, M.V. Lomonosov Moscow State University, Leninskie Gory 1, 119991 Moscow, Russia; akiushov1@yandex.ru (A.A.K.); zezinsb@yandex.ru (S.B.Z.); msa60@yandex.ru (M.S.A.); 2 Department of Soil Science, M.V. Lomonosov Moscow State University, Leninskie Gory 1, 119991 Moscow, Russia; dkhaydapova@yandex.ru

**Keywords:** polyelectrolytes, polycomplexes, amphiphilic polymers, clay, kaolinite, rheology

## Abstract

Wide usage of clay-based materials in industry requires investigations concerning efficient modification techniques to control their mechanical behavior in aqueous media. The challenging problem in this field involves minimization of the modifying agent content to provide marked changes in the operating characteristics of the material. In this work, the physicochemical, mechanical and structural aspects of the interaction of capillary water-saturated kaolinite with polyelectrolytes were studied. Modification of kaolinite with a negligible amount (0.1 wt.%) of hydrophilic and amphiphilic polyelectrolytes provides the control for rheological parameters of kaolinite suspensions such as storage and loss modulus in the range of three orders of magnitude. The results obtained reveal the wide possibilities for the production of a spectrum of clay materials using minor amounts of polymer modifying agents.

## 1. Introduction

Aluminosilicates—kaolinite, montmorillonite, bentonite, etc.—are widely used in composites and constructions, oil/gas, the paint and paper industry, cosmetology and pharmaceutics as well as materials for 3D printing and artwork [1,2,3,4,5,6,7]. However, well-pronounced water sorption by clay-based materials results in their structural transformations and marked changes in rheological behavior. In connection with this, efficient methods of modification of clays to control their physical and mechanical behavior in aqueous media seems to be of prime importance [8]. At the present time, polyelectrolytes (PEs) are considered as promising modifying agents for clay materials to control flocculation of clay colloids, loosening or stabilization of clay particles, swelling, clay dehydration, etc. [9]. The particular aspect of the problem involves minimization of the additive amount to attain the required operating parameters via structural modification of materials [10,11].

In this work, physicochemical and structural aspects of complexation of kaolinite with polyelectrolytes (PEs) are discussed to provide the efficient control for the rheological characteristics of the resulting material with the minor amount of modifier. The complexation was performed with cationic water-soluble poly (diallyldimethylammonium chloride) (PDADMAC) and anionic amphiphilic potassium humates (PHum), the latter contained condensed aromatic and heterocyclic rings and peripheral carboxylic groups [12]. The amphiphilic nature of humates determined their nano-sized compact structure with external anionic groups exposed to water. Additionally, inter-polyelectrolyte complexes, products of electrostatic interaction between PDADMAC and PHum, were used whose amphiphilicity was given by hydrophobic fragments represented by mutually neutralized PDADMAC-PHum blocks.

## 2. Materials and Methods

Commercial natural kaolinite (Sigma-Aldrich, St. Louis, MO, USA) was used for preparation of aqueous clay suspensions. Modification of kaolinite suspensions was carried out with the aqueous solutions in phosphate buffer (pH = 6.5) of anionic potassium humates (Mw = 9.9 kDa, Humintech GmbH, Grevenbroich, Germany) and cationic poly(diallyldimethylammonium chloride) (Mw = 200–350 kDa, Sigma-Aldrich) as well as negatively charged and positively charged inter-polyelectrolyte complexes (IPECs), products of cooperative electrostatic interaction between cationic PDADMAX and anionic PHum. The IPECs were prepared via mixing of aqueous PDADMAC and PHum solutions as described elsewhere [13].

To study the interaction of kaolinite with PHum, aqueous solutions of PHum with different concentration were added to 4 wt. % kaolinite suspensions in 0.005 M phosphate buffer (pH = 6.5). Kaolinite–PHum conjugates were centrifuged, and a content of PHum in solution was detected with a Hitachi UV 1240 spectrophotometer (Tokyo, Japan) at wavelength 350 nm. The amount of clay-bound PHum (A_PHum_) was calculated as the difference between PHum content in the virgin solution and in the solution after separation of kaolinite–PHum particles.

For kaolinite–PDADMAC systems, 2.5 mL of clay suspension (0.05 wt.%) was mixed with the different volumes of aqueous PDADMAC solution (0.013 wt.%). Electrophoretic mobility (EPM) of the particles was detected using Brookhaven Zeta Plus (Brookhaven Instruments, Holtsville, NY, USA).

X-ray diffraction measurements of dry kaolinite and kaolinite samples with 0.1 wt.% of PEs were carried out in a transmission mode using URD-6 diffractometer (Carl Zeiss, Oberkochen, Germany).

For mechanical testing, 3 g of air-dried kaolinite were saturated up to 78 wt.% with water (pH = 6.5), 0.125 wt.% aqueous solutions of individual polymer (PDADMAC or PHum), and 0.125 wt.% aqueous solutions of IPECs (pH = 6.5). The samples obtained were characterized by a 1 mg/g polymer–dry clay ratio. Rheology of the above samples was studied at 20 °C using a MCR-302 modular rheometer (Anton Paar, Austria) with a PP-25 parallel-plate measuring system in an amplitude sweep mode [14,15] at an angular frequency of 0.5 Hz in the strain range 0.001–100%.

## 3. Results and Discussion

**Figure 1:**
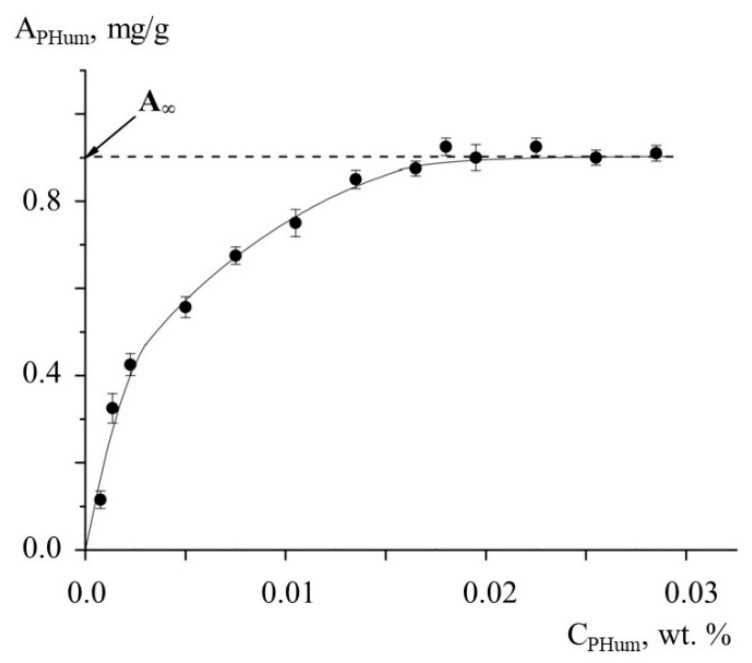
The dependences of the kaolinite-bound PHum, A_PHum_, on the concentration of aqueous PHum solutions, C_PHum_. 4 wt.% kaolinite aqueous suspension, pH = 6.5.

As the testing samples, the mixtures of kaolinite suspension with aqueous solutions of anionic potassium humates (PHum) and cationic poly(diallyldimethylammonium chloride) (PDADMAC) were used. The interaction between kaolinite and PEs was studied using the following procedures. For kaolinite–PHum samples, aqueous solutions of anionic PHum were added to an kaolinite aqueous suspension, clay–PHum conjugates were centrifuged, and a content of PHum in solution was detected spectrophotometrically. Figure 1 shows the dependence of the amount of clay-bound PHum (A_PHum_) on the concentration of PHum solutions, C_PHum_. With increasing PHum concentration, A_PHum_ approaches equilibrium value A_∞_ = 0.9 mg/g, which was considered as the kaolinite capacity to humates.

**Figure 2:**
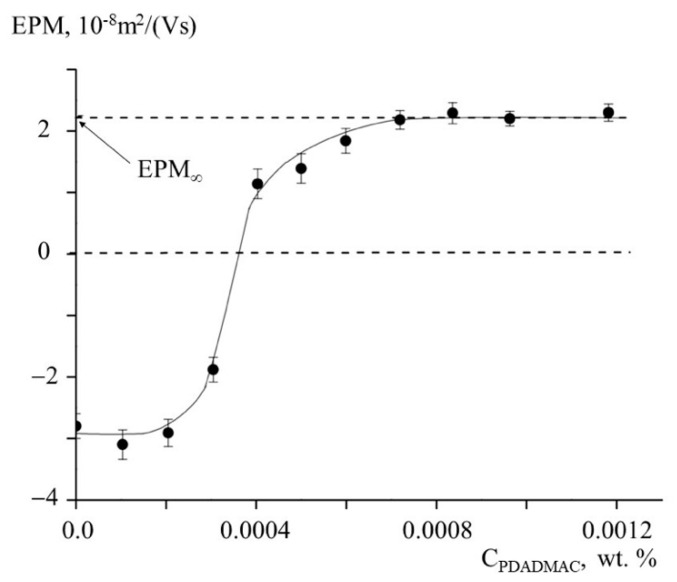
The dependences of electrophoretic mobility (EPM) of clay-PDADMAC suspensions on the concentration of aqueous PDADMAC solutions, C_PDADMAC_. 0.05 wt.% kaolinite aqueous suspension, pH = 6.5.

Kaolinite–PDADMAC samples were prepared by mixing of clay suspension and aqueous PDADMAC solution. In contrast to PHum, PDADMAC does not absorb in the visible spectrum. For this reason, a different approach for quantification of PDADMAC binding to the clay was used based on measuring electrophoretic mobility (EPM) of kaolinite–PDADMAC particles with laser microelectrophoresis. Figure 2 shows the dependence of EPM for kaolinite–PDADMAC particles (a parameter associated with their surface charge) on PDADMAC concentration C_PDADMAC_. With the introduction of cationic PDADMAC into a clay suspension, the charge on the anionic clay particles tends to zero, causing the charge neutralization at C_PDADMAC_ ~ 0.00035 wt. %. With the following increasing C_PDADMAC,_ the positive charge on the conjugate particles grows and approaches the equilibrium value EPM_∞_ at C_PDADMAC_ ~ 0.0007 wt. % which allowed us to estimate the capacity of kaolinite to PDADMAC as A_∞_ = 14 mg/g.

The above results provided the evidence of the interaction between kaolinite and both PEs. Note that the capacity of the clay for positively charged PDADMAC (14 mg/g) exceeded the corresponding value for negatively charged PHum (0.9 mg/g) for more than one order of magnitude. An effective binding of cationic PDADMAC to kaolinite has been noted previously by other researchers [16,17,18,19].

**Figure 3:**
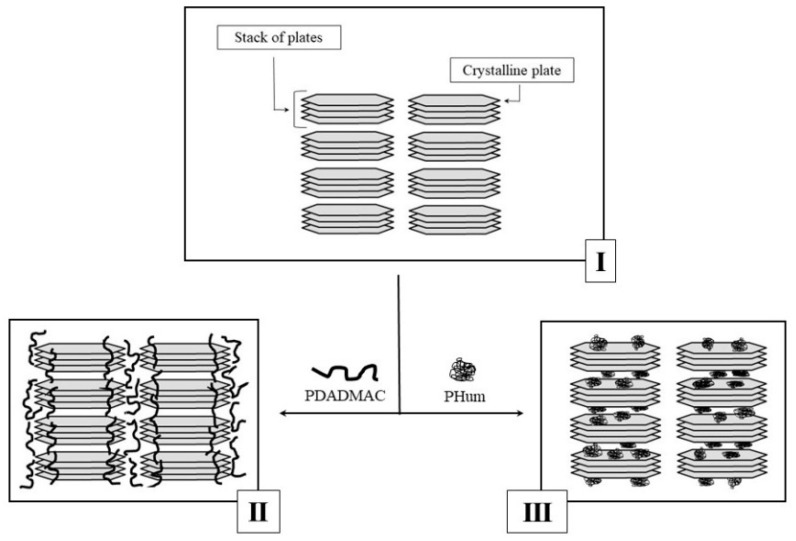
Schematic representation of the structure of virgin kaolinite (I), kaolinite–PDADMAC (II) and kaolinite–PHum samples (III).

To discuss structural aspects of the interaction of polyelectrolytes with kaolinite, let us mention that kaolinite is known to have a 1:1 sheet structure composed of SiO4 tetrahedral layers and Al(O,OH)6 octahedral layers with a diameter of ~10 μm and a thickness of 0.72 nm. A two-dimensional layer of silicate groups is bonded to a layer of aluminate groups [20]. These sheets form regular pseudo-hexagonal crystalline plates with the thickness of hundreds of nanometers, which are arranged in extended stack structure [21] (Figure 3, Scheme I).

In aqueous media, water penetrates between the stacks while inter-sheet and inter-plate spaces are practically inaccessible for water molecules. Polyelectrolyte macromolecules are able to absorb on the surface of the crystalline plates via electrostatic interactions for positively charged PDADMAC and via hydrogen bonding, ligand exchange, cation bridges and van der Waals interactions for negatively charged PHum [22].

These speculations allowed us to assess a kaolinite crystal area occupied by the cationic PDADMAC macromolecules in the saturated clay-polymer conjugate, *S_sut_*. The estimation was done from geometrical considerations based on the chemical formula of the polycation unit and the literature data for the lengths of chemical bonds in the unit [23]. Thus, the calculated area covered by a single PDADMAC monomer unit (*S_unit_* = 0.25 nm^2^) was then transformed into *S_sut_* as:

*S_sut_* = *N_A_* × *S_unit_* × *A_∞_/M* = 12.8 m^2^/g (1)

where *N_A_* is Avogadro’s number and *M* = 162 g/mole is a molecular mass of the PDADMAC unit.

The calculated value of *S_sut_* is in a good agreement with an overall surface area of kaolinite samples 20 ± 10 m^2^/g [24], assuming that in an ideal case the whole surface of the clay is covered with polyelectrolyte macromolecules. In reality, values of *S_sut_* seem to be smaller because of the flexibility of PDADMAC macromolecules responsible for the appearance of loops and tails when the polycation interacts with the negatively charged surface [9].

As for the anionic PHum, their macromolecules were shown to form colloid particles with the several dozens of nanometers in diameter when dispersed in a water medium [25,26]. For this reason, PHum is likely covered only a part of the kaolinite surface.

**Figure 4:**
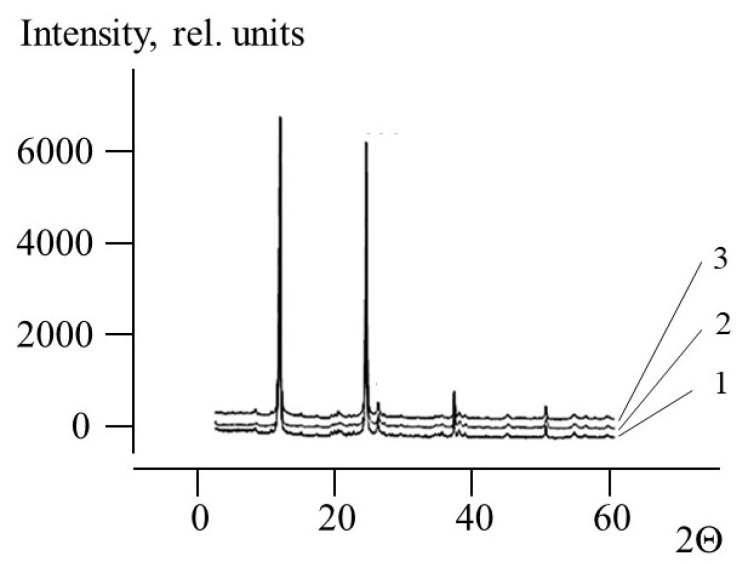
X-ray diffraction patterns of kaolinite (1), kaolinite–PHum (2), and kaolinite–PDADMAC dry samples (3).

For rheological testing kaolinite saturated with water as well as kaolinite saturated with aqueous solutions of PEs were used. In such equilibrated two-phase systems, aqueous media is known to be held in the clay due to capillary tension [27]. For all samples, the content of PEs was 1 mg/g (0.1 wt.%), which was comparable with the capacity of PHum to kaolinite (0.9 mg/g) and for one order of magnitude less than the capacity of PDADMAC (14 mg/g). For these samples, X-ray diffraction studies showed no shift of intrinsic reflections at 2θ = 12.481, 25.019, 26.767, and 37.858 Å (Figure 4) providing the evidence of no influence of polymers on the clay structure.

**Figure 5:**
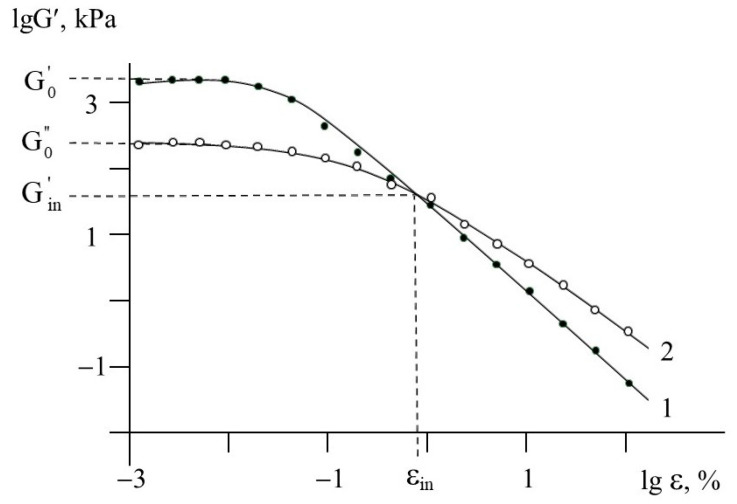
Strain dependences of storage modulus G^′^_0_ (1) and loss modulus G^″^_0_ (2) for kaolinite-PDADMAC sample. 78 wt.% kaolinite aqueous suspension, pH 6.5.

For the kaolinite–PDADMAC composite, Figure 5 shows the typical strain dependences of storage modulus G′ and loss modulus G″. When the strain increases, both G′ and G″ decrease due to mechanically stimulated destruction of the sample structure. At a certain strain ε_in_, the above dependencies intersect, providing the inversion of the elastic and viscous response of the sample. For all samples studied, the values of both initial storage modulus G^′^_0_ and initial loss modulus G^″^_0_ estimated at strain are not above 0.01 %, as well as the values of storage modulus G^′^_in_ and strain ε_in_ corresponding to the inversion point (Figure 5) are shown in Table 1.

The introduction of cationic PDADMAC to kaolinite results in a marked increase in the values of G^′^_0_, G^″^_0_ and G^′^_in_ and decrease in the value of ε_in_. The modification of clay with anionic PHum provides a dramatic decrease in the values of G^′^_0_, G^″^_0_ and G^′^_in_ and growth in the value of ε_in_. To vary the rheological characteristics within this interval, water-soluble non-stoichiometric inter-polyelectrolyte complexes (IPECs) were prepared via mixing of PHum and PDADMAC aqueous solutions [13] (Table 1). A negatively charged complex (IPEC(−)) was characterized by a 2-fold molar excess of anionic PHum groups over cationic PDADMAC groups, and a positively charged complex (IPEC(+)) by a 2-fold molar excess of cationic PDADMAC groups over anionic PHum groups. The solubility of both non-stoichiometric polycomplexes was due to excess charged PE units exposed into the aqueous phase.

Hence, the modification of kaolinite with a minor amount (0.1 wt.%) of ionic polymers allows one to control the rheological parameters for three orders of magnitude. The structural pattern of the above dramatic effect of a negligible (0.1 wt.%) amount of PEs on the rheological behavior of kaolinite is far from comprehension and requires further experimental studies. However, we suggested that the reinforcing action of cationic PDADMAC is associated with the preferential location of macromolecules on the edges of the kaolinite crystalline stacks (Figure 3, transition from Scheme I to Scheme II), where negative charges are concentrated [8,21]. These localized contacts result in the interconnection of individual stacks in the cooperative system. Colloid particles of anionic PHum occupy the inter-stack space (Figure 3, transition from Scheme I to Scheme III) and play the role of lubricant, which relieves the mutual slippage of stacks under shear deformation. Expectedly, a decrease in the content of the polymer, either PHum or PDADMAC, resulted in a pronounced mitigation of the polymer effect.

The positively charged IPEC(+) acts as a binder similar to the individual PDADMAC; the negatively charged IPEC(−) also demonstrates reinforcing action towards kaolinite, but is less pronounced than IPEC(+). As a whole, the action of IPECs with a controlled cationic-to-anionic ratio is the superposition of the effects produced by cationic and anionic polymers.

## 4. Conclusions

In summary, the usage of a negligible amount (0.1 wt.%) of two modifying agents (cationic PDADMAC and anionic PHum) as well as their complexes allows efficient control for rheological parameters of kaolinite in the range of three orders of magnitude. The mechanism of this pronounced influence of charged additives on rheological behavior of clay is likely to be associated with their preferential localization in structural sublevels at the edges of stacks and in inter-stack space, which are responsible for the shear deformation of the material. The results obtained reveal the wide possibilities to produce a spectrum of clay materials using minor amounts of a restricted range of modifying agents.

## Figures and Tables

**Table 1:** Rheological characteristics of kaolinite-polymer samples.

No.	Sample	$G_{0}^{\prime },\;\mathrm{kPa}$	$G_{0}^{\prime \prime },\;\mathrm{kPa}$	$G_{\mathrm{in}}^{\prime },\;\mathrm{kPa}$	ε_in, %_
1	Kaolinite	212.0 ± 10.0	70.0 ± 5.0	0.52 ± 0.04	9.8 ± 0.8
2	Kaolinite/PDADMAC	2080.0 ± 24.0	352.0 ± 69.0	115.0 ± 8.0	0.75 ± 0.14
3	Kaolinite/IPEC(+)	664.0 ± 62.0	182.0 ± 12.0	6.4 ± 0.5	3.8 ± 0.32
4	Kaolinite/IPEC(−)	410.0 ± 26.0	127.0 ± 10.0	1.67 ± 0.14	5.2 ± 0.3
5	Kaolinite/PHum	3.5 ± 0.8	0.67 ± 0.06	0.027 ± 0.003	73.6 ± 0.6

## Data Availability

All data are available in the main text.
